# Contrôle de qualité virologique du sang transfusé dans la ville de Bukavu, Sud Kivu, République Démocratique du Congo

**DOI:** 10.11604/pamj.2018.30.193.13457

**Published:** 2018-07-04

**Authors:** Théophile Mitima Kashosi, John Kivukuto Mutendela, David Lupande Mwenebitu, Jeff Kabinda Maotela, Kanigula Mubagwa

**Affiliations:** 1 Laboratoire de Recherche Biomédicale et de Santé Publique, Département de Sciences Biomédicales, Faculté de Médecine et Santé Communautaire, Université Évangélique en Afrique (UEA), République Démocratique du Congo; 2 Centre Internationale de Formation et de Recherches Avancées, Bukavu, République Démocratique du Congo; 3 Section Techniques de Laboratoire, Institut Supérieur des Techniques Médicales (ISTM) de Bukavu, République Démocratique du Congo; 4 Médecins d’Afrique, Coordination-Europe, Savigny Sur Orge, France; 5 Département de Biologie Clinique, Hôpital Provincial Général de Référence de Bukavu (HPGRB), Université Catholique de Bukavu, République Démocratique du Congo; 6 Centre Provincial de Transfusion Sanguine (CPTS) de Bukavu, Sud-Kivu, République Démocratique du Congo; 7 Département Scientifique Cardiovanculaire, Université de Leuven, Leuven, Belgique

**Keywords:** Transfusion, qualité virologique, Bukavu, République Démocratique du Congo, Transfusion, virological quality, Bukavu, Democratic Republic of Congo

## Abstract

**Introduction:**

Le sang transfusé à Bukavu est sélectionné par les tests rapides. Ces tests sont réalisés facilement sans moyens techniques importants. L'objectif de ce travail était d'évalué la qualité virologique du sang qualifié par les tests rapides (TDR).

**Méthodes:**

Pendant un mois, dans 5 formations sanitaires, un échantillon de sang était prélevé dans un tube sec de 4ml sur une poche de sang. Une contre analyse était faite sur chaque échantillon par des tests rapides et par Elisa. Les valeurs intrinsèques et extrinsèques ont été calculées. Le test Kappa de Cohen a été utilisé pour estimer la fiabilité des TDR par rapport à l'Elisa.

**Résultats:**

Trois cent douze échantillons ont été colligés; 5 échantillons étaient positifs à l'un ou l'autre marqueur virologique alors que 307 échantillons étaient négatifs à tous les tests. L'Elisa des 307 échantillons négatifs aux TDR avait révélé 15 autres prélèvements positifs dont 3 échantillons positifs au VIH, 3 au VHC et 9 pour le VHB. En plus, l'Elisa a confirmé certains cas positifs aux TDR et en a infirmé d'autres. La sensibilité et la valeur prédictive positive pour les tests de diagnostics rapides étaient très basses. La fiabilité de ces tests était satisfaisante, moyenne ou faible.

**Conclusion:**

Le sang qualifié par les TDR présente un risque infectieux viral non négligeable. L'usage des tests plus fiable et plus performant s'avère nécessaire dans notre milieu.

## Abstract (en)

**Introduction:**

In Bukavu, transfused blood is selected using rapid diagnostic tests (RDTs). These tests are easily performed without specialized equipments. This study aims to evaluate the virological quality of transfused blood assessed using rapid diagnostic tests.

**Methods:**

A blood sample was drawn from a blood bag and collected in a 4ml dry tube in 5 Health Care Facilities over a month. Counter analysis was performed on each sample using rapid tests and ELISA. Intrinsic and extrinsic values were calculated. Cohen’s kappa coefficient was used to evaluate the reliability of RDT compared with ELISA.

**Results:**

Three hundred and twelve samples were collected; 5 samples were positive for one or the other virologic marker while 307 samples were negative in all the tests. However Elisa showed, out of the 307 samples which were RDT test negative, 15 other positive samples including 3 samples positive for HIV, 3 for HCV and 9 for HBV. In addition, ELISA validated some RDT-positive samples and contradicted other results. Sensitivity and positive predictive value from rapid diagnostic tests were very low. The reliability of these tests was satisfactory, medium or low.

**Conclusion:**

Blood assessed using RDTs poses a non negligible risk of viral infections. This study highlights the need for more reliable and efficient tests in our Health Care Facilities.

## Introduction

Des milliers des vies humaines sont sauvées par la transfusion sanguine chaque année partout dans le monde. Cependant, celle-ci reste entachée de beaucoup des risques. Un risque résiduel transfusionnel persiste pour plusieurs agents infectieux [1, 2]. Dans les pays à faible revenu, chez les donneurs de sang, la prévalence de l'infection au Virus de l'Immunodéficience Humaine (VIH) se situe entre 1% et 12%, celle de l'hépatite C entre 0,5% et 12%, et celle de l'hépatite B entre 3% et 22%; les infections parasitaires curables telle que la malaria ne sont pas recherchées systématiquement [2]. Parmi les causes de la non-sécurité de la transfusion sanguine en Afrique Subsaharienne, on cite le manque de ressources matérielles pour tester et conserver le sang et le manque des ressources humaines suffisamment formées pour bien réaliser les analyses biologiques [2]. Plusieurs études sur la transfusion sanguine montrent un important risque résiduel infectieux caractérisé par l'utilisation des tests incapables de déceler les anticorps des virus durant la période de séroconversion [1-4]. Après plusieurs dons sanguins, certains donneurs se voient être diagnostiqués séropositifs pour l'un ou l'autre marqueur infectieux. Les receveurs courent ainsi le risque de contamination qui pourtant serait évité par l'utilisation des tests de dépistage de plus en plus performants.

En République Démocratique du Congo (RDC), chaque année, environs 9406 poches de sang prélevées chez les donneurs bénévoles sont transfusées. Ces poches sont qualifiées sur base des résultats des tests de diagnostics rapides (TDR) détectant certains anticorps viraux dans la majorité des structures sanitaires organisant la transfusion sauf dans quelques Centres Provinciaux de Transfusion Sanguine (CPTS) où existent des appareils pour les tests Elisa. Ainsi, environs 80% des poches sont sécurisées sauf pendant certaines périodes où sont observées des ruptures de stock des réactifs, liées entre autres au fait que les activités de transfusion soient souvent tributaires de financements extérieurs [5]. Au Sud-Kivu, le don de sang est organisé dans plusieurs formations sanitaires de manière autonome. Le circuit d'approvisionnement en réactifs de laboratoire n'est pas clairement défini. Faute d'un circuit formel, les structures sanitaires font recours aux fournisseurs privés dont le professionnalisme n'est pas garanti. Cette situation rend la sécurité transfusionnelle trop instable. Fort malheureusement, aucune mesure de contrôle de qualité n'est mise en place de manière permanente et régulière. C'est pourquoi l'objectif de ce travail était de réévalué la qualité du sang transfusé par les structures sanitaires à Bukavu en République Démocratique du Congo.

## Méthodes

**Type et site d'étude:** Une étude transversale a été organisée dans les cinq principales formations sanitaires de la ville de Bukavu, en Province du Sud-Kivu à l'Est de la République Démocratique du Congo (DRC) pendant le mois de mai 2015. Il s'agissait principalement de l'Hôpital Provincial Général de Référence de Bukavu (HPGRB), l'Hôpital Général de Référence de Panzi (HGRP), l'Hôpital Général de Référence de Ciriri (HGRC), l'Hôpital Général de Référence de Nyantende (HGRN) et le Centre Provincial de Transfusion Sanguine de Bukavu (CPTSB). Ces structures sont en effet, les principales formations sanitaires organisant le don du sang dans la ville de Bukavu et ses alentours. Hormis le « don passif », où un donneur s'amène volontairement à la structure sanitaire pour donner son sang et le don dit « familier » (où un membre de la famille du patient est invité à donner du sang en Urgence), ces structures sanitaires organisent régulièrement des campagnes de collecte du sang. Une équipe mobile se rend soit à l'église, soit dans un club (association) des donneurs de sang, soit dans les écoles d'enseignement supérieurs et universitaires. Là, l'équipe de transfusion sanguine sensibilise les personnes adultes à donner volontairement du sang aux malades internés dans leur hôpital; et seules quelques personnes, convaincues du message, peuvent passer à l'acte de don de sang. Ainsi, le sang est collecté directement dans une poche stérile citratée au niveau du site; mais la qualification biologique de la poche n'intervient qu'au laboratoire de chaque structure. Lors de chaque qualification biologique, les marqueurs infectieux recherchés systématiquement sont le Virus de l'Immunodéficience Humaine (VIH), le Virus de l'Hépatite B (VHB) et le Virus de l'Hépatite C (VHC). Parfois la syphilis est également recherchée mais pas systématiquement. Par la suite, une poche de sang qui présente un test de diagnostic positif ou douteux pour l'un des marqueurs infectieux est éliminée du don et sera détruite immédiatement tandis qu'une poche négative à tous les tests de diagnostics sera conservée au réfrigérateur et destinée à la transfusion ultérieure.

**Population d'étude:** Dans cette étude, la population était constituée des donneurs de sang. La classification des donneurs du sang (familial, bénévole régulier ou occasionnel) n'a pas été prise en compte. Le prélèvement de l'échantillon destiné à l'étude s'est effectué sur les poches de sang prélevées lors de campagne de sensibilisation ou lors d'un don à l'hôpital. Un aliquote de chaque échantillon biologique prélevé a servi au laboratoire de la structure sanitaire concernée pour la qualification de la poche et un autre était acheminé au laboratoire de recherche biomédicale et de santé publique de l'Université Evangélique en Afrique (dans une boite isotherme avec des vessies de glace) où l'évaluation de contrôle de qualité était effectuée.

**Analyses de Laboratoire:** Au Laboratoire, une contre-analyse était simultanément réalisée par des tests de diagnostics rapides (TDR) et le test Elisa. Les tests étaient faits sur du plasma obtenu après centrifugation à 3000 tour/minute pendant 5 minutes de l'échantillon. Le plasma ainsi obtenu était recueilli dans un tube Eppendorf de 2ml. Les 3 tests de diagnostics rapides utilisés étaient: Alere Determine' HIV-1/2, lot n°60965K100, référence n°7D2343; Cypress diagnostic HBsAg Dipstick, lot n°B201309013, référence n°142-050/S et Cypress Diagnostic anti-HCV Dipstick, lot n° B201407008, référence n°172-050/S respectivement pour la détection des anticorps du virus VIH, de l'hépatite B et de l'hépatite C. Aucun test de diagnostic rapide n'a été effectué pour la détection des anticorps de la syphilis. Les kits Elisa utilisés étaient respectivement ApDia Ag/Ab HIV 4^e^ génération pour le VIH, lot n°2013213, n° de référence 790001; Kit Elisa Dia source HBs Ag, lot n° B14906PT, n° de référence 20160302; Anti-HCV ELISA V4.0, lot n°C64C17PT, référence n°20160607 et DiaSource Syphilis IgG ELISA, lot n°A-065, référence n°KAPRSPG16. Les calculs des valeurs intrinsèques et extrinsèques des tous ces tests biologiques ont pris en compte les échantillons positifs et négatifs aux tests de diagnostics rapides.

**Analyse statistique des données:** Nous avions utilisé le logiciel Epi Info version 3.5.4, ainsi que le logiciel MedCalc software pour les différentes analyses statistiques. La sensibilité (Se), la spécificité (Sp), la valeur prédictive positive (VPP) et la valeur prédictive négative (VPN) ont été calculées pour déterminer les valeurs intrinsèques et extrinsèques des tests de diagnostics rapides (TDR) considérant le test Elisa comme le Gold standard. Le test Kappa de Cohen avait été utilisé pour évaluer la fiabilité des tests de diagnostics rapides. La grille de lecture proposée par Landis et Koch en 1977 avait été utilisée [6]: fiabilité excellente (K = 0,81-1,0), fiabilité satisfaisante (K=0,61-0,80), fiabilité moyenne (K = 0,41-0,60), faible fiabilité (K= 0,21-0,40). Le protocole de recherche avait été soumis et avait été agréé par le Comité d'éthique de l'Université Catholique de Bukavu (UCB) dans la lettre d'approbation numéro de référence UCB/CIE/NC/012/2015.

## Résultats

Au total, nous avions reçu 312 échantillons biologiques de sang de toutes les cinq structures sanitaires concernées. La contre-analyse par les tests de diagnostics rapides avait confirmé les résultats obtenus dans les structures d'étude: 5 échantillons étaient positifs à l'un et/ou l'autre marqueur viral. Le test Elisa avait été fait sur l'ensemble des échantillons: 307 prélèvements étaient négatifs pour tous marqueurs viraux. Notons cependant que l'Elisa avait révélé 3 échantillons positifs au VIH; 3 échantillons positifs au VHC et 9 échantillons positifs au VHB. L'Elisa a confirmé certains cas positifs aux tests de diagnostics rapides et en a infirmé d'autres (Tableau 1). La sensibilité était de 57,14% (18,41%-90,10%); 50,00% (11,81%-88,19%); 25,00% (5,49%-57,19%) pendant que la spécificité était de 99,67% (98,19%-99,99%); 99,35% (97,66%-99,92%); 99,33% (97,61%-99,92%) successivement pour les TDR VIH, VHC et VHB. Les valeurs extrinsèques de ces tests comme la VPP était de 80,00% (28,36%-99,49%) pour le test VIH et 60,00% (14,66%-94,73%) pour les test du VHC et du VHB. La VPN était de 99,02% (97,17%-99,80%) pour les tests du VIH et du VHC alors qu'elle était de 97,07% (94,51%-98,65%) pour le test du VHB. Le test Kappa cherchant l'accord entre les TDR et l'Elisa (variant entre 0 et 1) était successivement de 0.66 (0.55-0.76), 0.53 (0,42-0,64) et de 0,33 (0,23-0,43) pour le test du VIH, du VHC et du VHB (Tableau 2).

**Tableau 1:** Répartition des cas positifs à l’Elisa, négatif aux TDR selon les structures sanitaires

Elisa	HPGRB(n=76)	HGRP(n=65)	HGRC(n=63)	HGRN (n=47)	CPTSB (n=56)	TOT. (n=307)
HIV	1(1,32)	2(3,08)	0	0	0	3(0,97)
HCV	1(1,32)	0	1(1,59)	1(2,13)	0	3(0,97)
HBV	2(2,63)	4(6,35)	1(1,59)	0	2(3,57)	9(2,93)
Syphilis	0	1(1,54)	0	0	1(1,78)	2(0,65)

**Tableau 2:** Valeurs intrinsèques des TDR Alere Determine HIV1/2, Cypress diagnostics HBs Ag dipstick et Cypress diagnostic Anti-HCV dipstick par rapport à l’Elisa

Tests rapides		ELISA		Se(%)	Sp(%)	VPP(%)	VPN(%)	Kappa
		Positif	négatif	IC à 95%	IC à 95%	IC à 95%	IC à 95%	IC à 95%
VIH	Positif	4	1	57,14	99,67	80,00	99,02	0,66
	Négatif	3	304	18,41-90,10	98,19-99,99	28,36-99,49	97,17-99,80	0,55-0,76
VHC	Positif	3	2	50,00	99,35	60,00	99,02	0,53
	Négatif	3	304	11,81-88,19	97,66-99,92	14,66-94,73	97,17-99,80	0,42-0,64
	Positif	3	2	25,00	99,33	60,00	97,07	0,33
VHB	Négatif	9	298	5,49-57,19	97,61-99,92	14,66-94,73	94,51-98,65	0,23-0,43

## Discussion

**Taux d'infections virales dans les échantillons à tests de diagnostics rapides négatifs:** Le risque de transmission d'agents infectieux reste l'une préoccupation majeure lors d'une transfusion sanguine. Nos résultats ont montré que le sang sélectionné à l'aide des tests de diagnostics rapides (TDR) présente un taux de 0,97% pour les virus d'immunodéficience humaine et de l'hépatite C et de 2,93% pour le virus de l'hépatite B. En effet, les qualités intrinsèques des tests de diagnostics rapides restent limitées avec une faible sensibilité diagnostique dans les phases précoces de l'infection [7]. Tous les tests de diagnostics rapides utilisés à Bukavu sont basés uniquement sur la détection d'anticorps viraux pour le VIH et le VHC. Les donneurs se retrouvant dans la période de séroconversion ou ceux ayant un taux d'anticorps non détectables ne peuvent donc pas être détectés par ces types des tests. Au Mali [8], une étude menée par Diarra A et al. en 2009 avaient trouvé des prévalences plus élevées dans les dons de sang (2,6% pour le HIV, 3,3% pour le HCV, 13.9% pour l'antigène HBs). Dans cette étude, la prévalence d'infections virales était plus forte chez les donneurs occasionnels que chez les donneurs réguliers. Notre étude n'a pas spécifié le type de dons, dans l'idée que dans la transfusion sanguine, les tests diagnostiques à utiliser devraient être les plus sensibles possibles de manière à minimiser tout risque infectieux, et ce, quel que soit les conditions du don de sang. La différence entre notre étude et celle de Diarra et al. est le nombre d'échantillons analysés (25543) et la longue période de recrutement des donneurs de sang (1 an) dans l'étude menée par Diarra et al. En Tunisie, en 2007, dans une étude portant sur le risque infectieux menée chez les polytransfusés, Hannachi N et al. avaient trouvé une prévalence de 8,4% pour l'antigène HBs, 4,7% pour le HCV et 0,86% pour le HIV; une différence statistiquement significative avait été notée dans cette étude entre les polytransfusés et les patients étiquetés non polytransfusés [1]. Cette étude prouve le risque infectieux que courent les patients polytransfusés dans le contexte socio-économique défavorisé. Etant donné qu'aucun test diagnostique n'est fiable à 100%, la transfusion sanguine reste toujours entachée des risques infectieux résiduels; mais des mesures visant à réduire le plus possible ce risque devraient être préconisées. Dans le dépistage du VIH, des tests diagnostiques détectant en même temps l'antigène p24, d'autres antigènes de VIH ainsi que des anticorps anti-VIH existent et sont recommandés en pareille circonstance [9].

Une étude réalisée au Gabon par Ndjoyi et al. en 2005 sur l'évaluation de 9 trousses de dépistage du VIH avait montré qu'il est impossible d'établir un diagnostic sérologique fiable du VIH avec une seule trousse de dépistage [10]. Le Centers for Disease Control and Prevention (CDC) avait recommandé depuis Juin 2014, l'usage (dans la stratégie 1 de diagnostic du VIH) d'un test basé sur la détection des anticorps, d'antigènes et de la protéine p.24 pour le diagnostic du VIH [9]. En RDC, particulièrement à Bukavu dans la province du Sud-Kivu, la sélection et la qualification virologique du sang pour le VIH est faite par un seul test de diagnostic rapide (TDR) basé sur la détection des anticorps [5]. Dans leur étude sur la prévalence et incidence du VIH et de l'hépatite B chez les donneurs de sang et sur l'estimation du risque résiduel de transmission du VIH et du virus VHB par la transfusion sanguine à Bukavu en 2009, Namululi et al. avaient trouvé une incidence de 3,57/1000 personnes-années pour le VIH et 25,4/1000 personnes-année avec un risque résiduel de 0,22 et de 3,9 successivement pour les deux virus [2]. En 2014, dans cette même ville, Kabinda et al. dans une étude sur les facteurs associés aux infections virales chez les donneurs de sang, avaient noté une prévalence de 4,8% d'hépatite B, 3,9% d'hépatite C et 1,6% de VIH chez les donneurs [4]. Ils avaient également retenu un risque résiduel de la transfusion sanguine de 0,6/1000 dons, 3,1/1000 dons, 7,9/1000 dons successivement pour le HIV, HCV et l'antigène HBs [4]. Nos résultats rencontrent ceux trouvés dans cette ville sur le risque viral résiduel de la transfusion sanguine; l'usage des tests de diagnostics rapides détectant seulement les anticorps occuperait une place de choix parmi les facteurs favorisant ce risque. De tous ces résultats, rappelons que la sécurité transfusionnelle devrait être renforcée davantage à Bukavu par l'utilisation des tests de diagnostics plus sensibles pouvant diagnostiquer les 3 infections virales ciblées même pendant la primo-infection, voire plus précocement.

**Valeurs intrinsèques des tests de diagnostics rapides (TDR) en transfusion sanguine:** Nos résultats ont montré une faible sensibilité diagnostique des TDR par rapport au test Elisa (57,14%; 50,00%; 25,00%) successivement pour le VIH, VHC et VHB. Ces résultats sont similaires à ceux trouvés en Ethiopie par Dessie A. et al. qui avaient rapporté une sensibilité faible du Determine HIV-1/2 de 60,5% [11] et ceux publiés en France en 2008 par l'agence Française de sécurité sanitaire des produits de santé (AFSSAPS) qui avait trouvé une sensibilité moindre des tests de diagnostics rapides surtout sur le panel de séroconversion [12]. En revanche, nos résultats sont en contradiction avec plusieurs autres études Africaines montrant une sensibilité et spécificité relativement élevées [10,13-15]. Mais, dans la plus part de ces études, les échantillons biologiques semblent issus des personnes malades évolutifs probablement ayant déjà un taux d'anticorps élevé. Le diagnostic de l'infection VIH est initialement basé sur la détection des anticorps anti-VIH. Cependant, durant la période dite de fenêtre sérologique, certains tests sont incapables de dépister la maladie. Les tests assurant la détection de l'antigène p24 et/ou des acides nucléiques viraux permettent ainsi de réduire la durée de cette fenêtre sérologique. Ces genres des tests seraient bien l'idéal en transfusion sanguine dans notre milieu. L'étude menée par S. Yerly et ses collaborateurs en France montre que l'utilisation des tests assurant la détection de l'antigène p24 et/ou des acides nucléiques du VIH permettait le diagnostic précoce des primo-infections à VIH [16]. En France le recours aux tests de diagnostics rapides (TDR) ne se justifie qu'en cas d'usage individuel ou en cas d'urgence (accident professionnel d'exposition au sang; accident d'exposition sexuel; accouchement chez les femmes enceintes dont le statut sérologique par rapport au VIH n'est pas connu, ou chez les femmes enceintes ayant eu une exposition supposée au VIH depuis la réalisation du dernier test de dépistage au cours de la grossesse; urgence diagnostique devant la survenue d'une pathologie aiguë évocatrice du stade Sida) afin de pouvoir mettre en œuvre rapidement une prise en charge médicale adaptée [15].

En Côte d'Ivoire, comme dans la majorité des pays Africains, le test Elisa est fait de manière systématique sur toutes les poches de sang seulement au niveau de la capitale. Mais à l'intérieur du pays, seul le test de diagnostic rapide est effectué sur seulement 1/3 des poches transfusés [5,17-18]. Ainsi les receveurs courent le risque de se voir contaminé à l'occasion de la transfusion. N. Hannachi dans son étude menée en Tunisie chez les polytransfusés avait trouvés qu'entre autres facteurs du risque de contamination chez les polytransfusés existait l'absence de détection du virus par les techniques standard de dépistage sérologique [1]. Dans cette même étude, il note un grand risque de contamination par les virus des hépatites (VHB et VHC). Notre étude et ceux d'autres collègues africains montrent que les virus des hépatites constituent une menace réelle pour les receveurs du sang [19-23], surtout lorsque ce dernier est sélectionné par des tests de diagnostics rapides. Touré-Fall AO et ses collaborateurs de Dakar au Sénégal avaient également trouvé un risque viral résiduel de la transfusion sanguine élevé pour le VHB et le VIH respectivement de 138/100000 dons et de 3,5/100000 dons pour une période de trois ans. Ils recommandent pour diminuer le plus possible ce risque l'introduction des méthodes de diagnostics plus sensibles comme la détection du génome viral dans la sécurité transfusionnelle [3]. La réglementation Française dans le dépistage du VIH exige actuellement l'utilisation d'un test Elisa unique qui doit permettre la détection combinée des anticorps anti-VIH-1 et 2 et de l'antigène p24 du VIH-1 avec un seuil minimal de détection de l'antigène p24 du VIH-1 de deux unités internationales (UI) par millilitre d'échantillon sérique ou plasmatique [15].

Enfin disons que les TDR présentent tout de même plusieurs avantages dont une bonne facilité d'utilisation, la possibilité de stockage à température ambiante et la possibilité de le réaliser en tout lieu. Ils possèdent une bonne sensibilité et spécificité pendant la phase chronique de l'infection [7,15]. Dans les pays à ressources limitées, ces tests ont révolutionnés le dépistage du VIH et n'exigent ni du personnel très qualifié ni des équipements très lourds. En revanche, les TDR sont entachés de quelques inconvénients comme le manque de sensibilité en phase précoce de l'infection, une lecture des résultats parfois subjective, des prix généralement élevé [7,15]. Actuellement, certaines études montrent la limites des tests de diagnostics rapides même ceux de 4^e^ génération dans le diagnostic du VIH [24-27]. Une mauvaise procédure de test sur le terrain peut conduire à des niveaux extrêmement faibles de sensibilité rapide au test VIH. Certaines marques de kits de tests de diagnostics rapides peuvent avoir de meilleurs résultats que d´autres lorsqu´elles sont confrontées à une utilisation sous-optimale. Ces incommodités rendent une fois de plus impératif que des mesures rigoureuses de contrôle de la qualité soient mises en œuvre là où elles n´existent pas et ne réservent ces tests de diagnostics rapides que dans certaines circonstances autre que la transfusion.

## Conclusion

Le sang sélectionné par les tests de diagnostics rapides (TDR) présente un risque infectieux viral important en République Démocratique du Congo et particulièrement à Bukavu. Compte tenu de leur faible sensibilité et spécificité, les TDR devraient être évités dans la sécurité transfusionnelle. L'usage des tests plus sensibles et la mise en place d'un système d'assurance et de contrôle de qualité régulier et permanent seraient un atout pour assurer et maintenir une meilleure qualité virologique du sang transfusé en Province du Sud-Kivu.

### Etat des connaissances actuelle sur le sujet

- Les tests de diagnostic rapides (TDR) sont sensibles et spécifiques dans le diagnostic des plusieurs maladies infectieuses;
- La transfusion sanguine est entachée d'un risque résiduel de transmission de certaines maladies comme le VIH, l'Hépatite B et l'hépatite C;
- Notre étude prouve la nécessité d'utiliser des tests plus sensibles comme la PCR dans le contexte de la transfusion sanguine.

### Contribution de notre étude à la connaissance

- Notre étude montre le risque virologique du sang sélectionné par des tests rapides;
- Notre étude montre les valeurs intrinsèques et extrinsèques des différents tests rapides dans le contexte de la transfusion sanguine à Bukavu, Est de la République Démocratique du Congo;
- Notre étude prouve la nécessité d'utiliser des tests plus sensibles comme la PCR dans le contexte de la transfusion sanguine.

## Conflits d’intérêts

Les auteurs ne déclarent aucun conflit d'intérêt.
